# The Hanbury Medallist

**Published:** 1915-07-24

**Authors:** 


### The Hanbury Medallist.

### A GREAT PHARMACOLOGIST AND HIS WORK.

The Hanbury Medal, which is awarded biennially for high excellence in the prosecution or promotion of original research in the chemistry or natural history of drugs, has this year been adjudicated to Mr. E. M. Holmes, F.L.S., who has been curator of the Pharmaceutical Society's museums for nearly half a century. Mr. Holmes is only the fourth Englishman to whom the medal has been awarded, the coveted prize having most frequently gone to a Continental scientist. It is perhaps not without interest to mention that Professor Emil Schmidt, of Marburg, the winner of the medal a few years ago, presented it, shortly after the outbreak of war, to the German Red Cross Society to be realised for the funds; what the medal sold for is not known, but the gold of which it is made was valued at £25. Mr. Holmes, who is frequently consulted by pharmaceutical and medical authorities in many parts of the world, is recognised as a great pharmacognosist, and as a medical botanist he has perhaps no equal. He was the first to whom the award was made of the medal struck in memory of the great botanist Flückiger, and he is the only recipient of the two medals. For the British Pharmacopœias of 1898 and 1914 he acted as botanical referee in conjunction with the Director of Kew Gardens; in connection with the cultivation of medicinal plants he has done extremely valuable work.

